# Dichromatic Colour Vision in Wallabies as Characterised by Three Behavioural Paradigms

**DOI:** 10.1371/journal.pone.0086531

**Published:** 2014-01-29

**Authors:** Wiebke Ebeling, Jan M. Hemmi

**Affiliations:** 1 Curtin Institute of Radio Astronomy, Curtin University, Bentley, Western Australia, Australia; 2 School of Animal Biology & The UWA Oceans Institute, The University of Western Australia, Crawley, Western Australia, Australia; Lund University, Sweden

## Abstract

Despite lacking genetic evidence of a third cone opsin in the retina of any Australian marsupial, most species tested so far appear to be trichromatic. In the light of this, we have re-examined colour vision of the tammar wallaby which had previously been identified as a dichromat. Three different psychophysical tests, based on an operant conditioning paradigm, were used to confirm that colour perception in the wallaby can be predicted and conclusively explained by the existence of only two cone types. Firstly, colour-mixing experiments revealed a Confusion Point between the three primary colours of a LCD monitor that can be predicted by the cone excitation ratio of the short- and middle-wavelength sensitive cones. Secondly, the wavelength discrimination ability in the wallaby, when tested with monochromatic stimuli, was found to be limited to a narrow range between 440 nm and 500 nm. Lastly, an experiment designed to test the wallaby’s ability to discriminate monochromatic lights from a white light provided clear evidence for a Neutral Point around 485 nm where discrimination consistently failed. Relative colour discrimination seemed clearly preferred but it was possible to train a wallaby to perform absolute colour discriminations. The results confirm the tammar wallaby as a dichromat, and so far the only behaviourally confirmed dichromat among the Australian marsupials.

## Introduction

Marsupials have presented us with an unusual case of diversity in mammalian colour vision, apparently having evolved both dichromatic and trichromatic species but lacking evidence of a third photopigment in the latter. Dichromatic colour vision is based on comparing the responses of two spectrally distinct cone types whereas trichromatic colour vision is based on three different cone types. Dichromacy was generally assumed to be the dominant and ancestral mammalian colour vision system as most mammals have been found to express a short-wavelength sensitive pigment in S-cones and a middle-to-long-wavelength sensitive pigment in M-cones [1]. Early investigations in American marsupials (Virginia opossum [2]), later supplemented by histological experiments [3] and by research in Australian marsupials (tammar wallaby [4], [5], [6]), supported this prevailing viewpoint that all mammals, other than primates – who possess three distinct pigments –, are dichromats. A number of more recent publications, however, have suggested the presence of a third cone type in the retinae of several Australian marsupials, namely the fat-tailed dunnart [7]–[9], honey possum [7], [8], bandicoot or quenda [10], and quokka [10]. Of these species, the dunnart was the only one tested in behavioural experiments and demonstrated to be able of making trichromatic colour discriminations [11]. Intriguingly though, we are still lacking evidence of the gene that gives rise to this third cone type [9], [12]–[15].

The tammar wallaby is so far the only member of the Australian marsupials studied that has not shown any evidence of trichromacy, although it is a close relative to the supposedly trichromatic quokka [10]. In the wallaby, data from immunohistochemistry, physiology, and behaviour are all in agreement with dichromatic colour vision [4]–[6], [9]. The present behavioural study was designed to re-address the question of dichromatic or trichromatic colour vision in the wallaby by means of monochromatic test lights in standardised colour vision tests, similar to studies in other mammalian species, to rule out the existence of a small population of previously undetected third cone type. Our results confirm the wallaby as a dichromat.

## Materials and Methods

### Animals

Data presented in this paper were collected between February 2008 and February 2010. All experimental procedures were approved by the Animal Experimentation Ethics Committee of The Australian National University (permits R.VS.21.06 and R.VS.26.08 to W. Ebeling). Three young adult tammar wallabies (*Macropus eugenii*), two males (Miller and Boris; approx. 9.5 kg) and one female (Kiwi, approx. 6 kg), were sourced from a breeding colony at The Australian National University (permit R.SD.05.07 to L. Marotte). Experiments took place in outdoor enclosures in which the test animals were fenced off from neighbouring wallabies by mesh wire. While test animals had *ad libitum* access to grass, hay, and fresh water, the experiments provided the only source of solid food (Ø5 mm horse pellets; Y.S. Feeds Pty Ltd, Young, New South Wales, Australia). Animals were only handled for transfers between enclosures and occasional medical checks. Running automated experiments at night minimised observer interference and allowed the animals to work during the night when they are naturally more active. In order to keep external illumination conditions as constant as possible, experiments always started after sunset and stopped before sunrise. Within this period, animals were free to start, pause, resume, and finish participating, with the exception of a 30-minute power-down interval for the monochromatic light sources in case the wallaby had been inactive for at least 30 minutes.

### Experimental Setup

**Figure 1:**
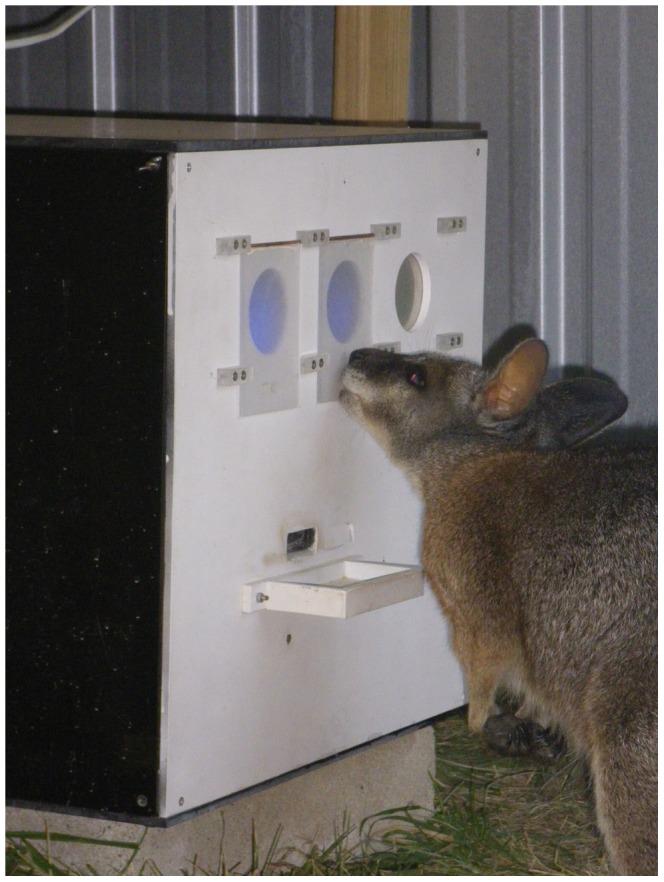
Wallabies readily used the automated operant conditioning setup for colour vision experiments. Light stimuli were projected onto diffuser flaps that also served as the trigger when the animal pushed to indicate a stimulus choice. If correct, a food reward was delivered into a feeder bowl under the stimuli. Photo copyright: W. Ebeling.

A custom-made operant conditioning apparatus, similar to the one described in [4], was used for all experiments (Figure 1). The setup was fully automated and could be remotely controlled via network connection. Experiments could be supervised in real-time using webcams. The apparatus was positioned in the wallabies’ outdoor enclosures, either under a tarp cover (Experiment 1) or inside a small shed (dimensions: L 2260 mm×W 1500 mm×H 1800 mm; Experiments 2 and 3). Experiment 1 was conducted in the dark. Experiments 2 and 3 were conducted under ambient light (1.6×10^−6^ W/cm^2^ sr at the position of the stimuli), provided by two fluorescent light tubes (Crompton Lighting, Sydney, New South Wales, Australia) that were mounted inside the shed. Visual stimuli (Ø50 mm) were back-projected at approximately wallaby eye height onto two transparent panels separated by gap of 10.5 mm (Experiment 1) or 50 mm (Experiments 2 and 3; Figure 1) width. Animals indicated their stimulus choice by pressing against one of the panels, thereby triggering the micro-switch underneath. As a reward mechanism, food pellets were delivered into a small feeder bowl below the stimulus panels such that the animal had to lower its head and take its eyes off the stimuli while retrieving the reward.

### Operant Conditioning

All experiments were based on a two-alternative forced-choice paradigm with positive reinforcement. Animals were initially trained to choose a positive white over a negative black stimulus. Once stimulus discrimination was reliable, coloured stimuli were introduced. The specific reward rules for particular stimulus pairs varied with experiments (see below). During training, a correct response was always rewarded with a short beep and a food reward whereas an incorrect response triggered a short timeout (3–15 seconds) during which time the stimulus lights were switched off and the apparatus became unresponsive. To counter-act a potential position bias, easy discriminations were repeated until the animal chose correctly, thus forcing the animals to regularly switch sides, but only the first choice for each stimulus combination counted towards the results. To ensure animals were not frustrated by poor performance, difficult discriminations were not repeated until a correct response was given. The aim was to keep the overall performance above 70% correct for first responses, and discrimination performance was continuously monitored for judgement of easy and difficult discriminations. In addition, some stimulus pairs were only tested but not trained (‘transfer trials’). For such transfer trials, animals did not receive feedback on their choice (no beep, no food, and no timeout), and the next stimulus pair was displayed immediately. Transfer trials were interspersed between normal trials at a default rate of one in every six trials, unless specified otherwise.

### Experimental Design

For all experiments, stimulus presentation was based on a randomised block design that included equal numbers of presentations of all training stimulus pairs. Transfer trials were randomised separately and then inserted at the appropriate intervals into the training trials. Within each block, each stimulus pair was presented twice, once as left-positive and once as right-positive in order to balance against any spatial bias that the animals might have had. To prevent the wallabies from switching to a spatial strategy, the left/right position of the positive stimulus was randomised with the restriction that the positive stimulus was never shown on the same side more than three consecutive times.

### Light Sources and Calibrations

**Figure 2:**
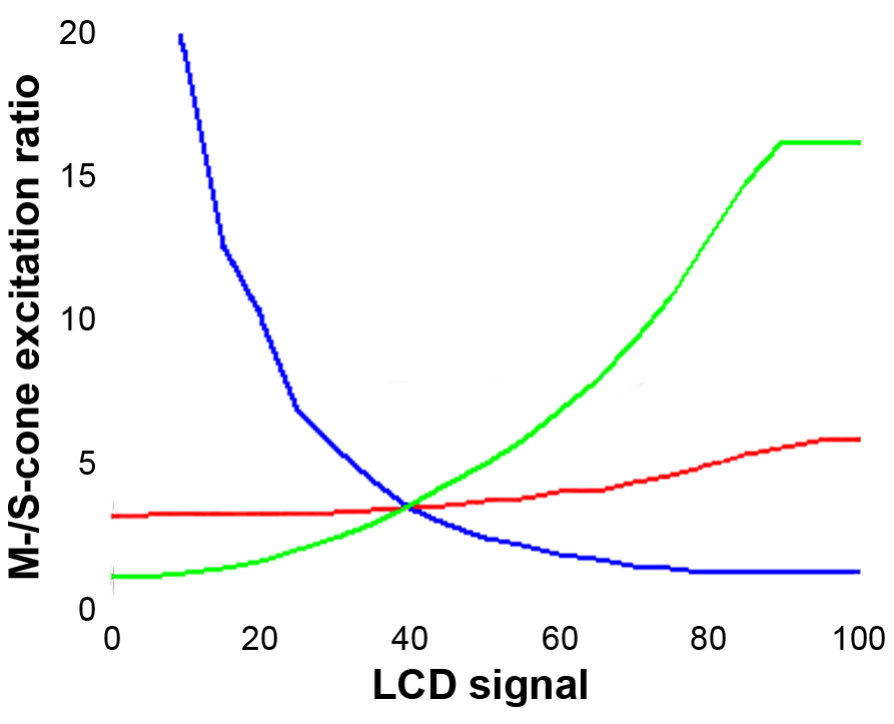
Conversion of measured LCD signals into M−/S-cone excitation ratios. Calibration of the three primary channels of the LCD monitor – red, green, and blue – made it possible to estimate the response strengths of M- and S-cones to any given colour signal and calculate the M−/S-cone excitation ratio.

For the LCD monitor used in Experiment 1 (LCD Colour-Mixing), light intensity of the colour stimuli were measured using a radiance sensor (ILT1700; International Lights, Peabody, Massachusetts, USA). The spectral sensitivity of the device was calibrated against a calibrated USB2000+ fibre optics spectrometer (Ocean Optics, Dunedin, Florida, USA). Spectral measurements were obtained from the inner ^2^/_3_ of a circular stimulus window by measuring from a distance of 50 mm. This procedure allowed us to convert the intensity readings into relative photon counts and to calculate the relative stimulus intensities as seen by different visual pigments with known spectral sensitivity. From this it was possible to estimate relative excitation ratios for different cone types (Figure 2). As there is no spectral luminosity function available for the wallaby and the exact ratio of S- to M-cones is not known, we estimated perceived intensity to be dominated by the M-cones [5], [6], [9] and varied the absolute and relative intensity of the light stimuli.

Monochromatic lights of approximately 15 nm bandwidth (less for wavelengths above 480 nm) were generated by two xenon light sources with integrated monochromators (Polychrome V; TILL Photonics GmbH, Gräfelfing, Bavaria, Germany) and fed through a Ø1.1 mm optical fibre and a BK7 glass diffuser (double-frosted W 60 mm×H 60 mm×T 1 mm; Tempotec Optics, Fuzhou, Fujian, China) onto the stimulus panels (single-frosted BK7 glass, W 70 mm×H 100 mm×T 3 mm). The light output of the two monochromators used in Experiment 2 (Wavelength Discrimination) was matched by scaling according to the absolute peak intensity reached. For Experiment 3 (Neutral Point), one of the two monochromators was replaced by the combined output of two white light sources (Halogen ‘Mikropack’ HL-2000-FHSA 24 V and LS-1 tungsten halogen 12 V; Ocean Optics, Dunedin, Florida, USA). The intensity of the white light source in this experiment could not be adapted dynamically and was always shown at its maximum intensity. The intensity of the monochromator-generated colour stimuli, therefore, was matched to the white using the relative stimulus intensity as seen by a 540 nm pigment. For calibration of the monochromators, an irradiance detector on the ILT1700 was used in combination with a flat response filter (International Lights, Peabody, Massachusetts, USA). Measurements were taken through a black tube at 200 mm distance from the panel, resulting in an aperture angle of approx. 7 degrees. These measurements were scaled according to the spectrograph’s spectral sensitivity. To ensure that the animal was not trained to use intensity as a cue where the monochromatic stimuli were not perfectly intensity-matched to the white, colours were presented at 50%, 75%, 100%, 150% or 200% of the intensity of the white light. Exceptions from this were as follows: 450 nm could only be produced at matched intensity, and 460 nm could only be shown at 75%, 100%, and 150% intensity.

### Experiment 1: LCD Colour-Mixing

The aim of this experiment was to find out whether one of the three primary LCD colours could be matched by a combination of the other two colours. Each colour produces a certain cone excitation ration which underlies colour perception. As colours of the same cone excitation ratio are indistinguishable for the animal, matching a particular excitation ratio leads to a Confusion Point. In case all such matches can be achieved with only two colours, such a Confusion Point is indicative of the one-dimensional colour space (excluding brightness) of a dichromat, whereas three colours have to be combined to match all colours in trichromatic species [16]. For example, the effect of adding a long-wavelength light to a grey light can, therefore, be mimicked by removing some of the blue light from grey for dichromats.

Stimuli were presented on a LCD monitor (740B, Samsung, Seoul, South Korea), placed flush against a black board with circular Ø50 mm cut-outs just behind the two stimulus panels (single-frosted Perspex, W 70 mm×H 100 mm×T 3 mm).

For all experimental constellations (Tests 1, 2, and 3; see below), the three animals were trained in a basic relative colour discrimination task by rewarding choice of colours with a higher estimated M/S-cone excitation ratio. For instance, a ratio of x implies the x-times stronger excitation of M-cones than of S-cones. Red stimuli (RGB percentage values [80/40/40], [70/40/40], and [60/40/40]) were rewarded over blue stimuli (RGB = [40/40/80], [40/40/70], and [40/40/60], respectively) and grey stimuli (RGB = [40/40/40]). The choice of green (RGB = [40/80/40], [40/70/40], and [40/60/40]) was rewarded over blue (RGB = [40/40/80], [40/40/70], and [40/40/60], respectively). To test the Confusion Points between the three primary channels of the LCD monitor, transfer trials were included at a rate of one every six to ten trials. Test 1 compared a red (RGB = [80/40/40]) against a grey stimulus (RGB = [40/40/40]) with an increasing contribution of green. The question was whether the wallabies would ever confuse the green and the red stimuli, and how much green would have to be added to the grey for this to happen. The RGB values for the green stimulus ranged from RGB = [40/40/40] to [40/65/40] for wallaby Miller or from RGB = [40/40/40] to [40/58/40] for Boris and Kiwi. Test 2 compared the same red (RGB = [80/40/40]) against a grey (RGB = [40/40/40]) with decreasing amounts of blue contribution (from RGB = [40/40/40] to [40/40/05] for Miller or from RGB = [40/40/40] to [40/40/20] for Boris and Kiwi). Test 3 was only carried out with wallaby Miller as the monitor had suffered thunderstorm damage and could not be replaced in time to continue experiments with wallabies Boris and Kiwi. This test compared a green (RGB = [40/65/40]) against a grey stimulus (RGB = [40/40/40]) with a decreasing blue component from RGB = [40/40/35]. The prediction for all three tests was that a Confusion Point existed where the wallabies would not be able to distinguish between the two differently coloured lights and that there would be a reversal of preference, leading the wallabies to avoid the colour that was preferred during training. The Confusion Point was determined as the 50% performance mark intersecting with a second order polynomial interpolation of the data points. Across all stimuli presented in these three tests, stimulus brightness varied between 1.2–2×10^−4^ W/cm^2^sr such that there was no consistent cue that would have allowed the animals to base their choices on brightness.

Cone responses were calculated according to:
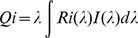
with *i* = 2 for a dichromatic system, *Qi* = quantum catch of receptor *i*, *Ri(λ)* = spectral sensitivity of receptor *i*, *I(λ)* = spectral distribution of light stimulus, integrating over the visible spectrum [16], [17].

### Experiment 2: Wavelength Discrimination

In this experiment, also called ‘Minimum Delta Lambda’, the wallaby’s ability to discriminate two wavelengths across a range of wavelengths was tested as the shape and extent of the discrimination space is indicative of how many spectrally distinct photoreceptors contribute to colour perception. Spectral discrimination ability was determined for seven so-called ‘centre wavelengths’ between 420 nm and 540 nm at 20 nm intervals. Intensities at these wavelengths were matched based on M-cone intensity (assuming a 540 nm pigment) but always presented at 40% Michelson contrast (i.e. difference over the sum) to mitigate effects of mismatching. We interspersed non-reinforced intensity tests at a rate of one in every 20 trials, presenting the centre wavelength at 40% contrast against itself, to ensure stimulus choice was not based on stimulus intensity. In all other stimulus pairs, however, the centre wavelength was never shown but instead the centre wavelength +/−16 nm, 8 nm, 4 nm, 2 nm, and 1 nm were compared. This is equivalent to wavelength differences (Δλ) of 32 nm, 16 nm, 8 nm, 4 nm, and 2 nm, respectively. Choice frequencies were plotted against the respective centre wavelength. Wallaby Boris was initially trained to a relative wavelength discrimination task, being rewarded for choosing the monochromatic colour with the longer wavelength for centre wavelengths 460 nm, 480 nm, and 500 nm at Δλ = 32 nm. The training regime was adjusted dynamically for other centre wavelengths and for other Δλ values as soon as performance at the specific centre wavelength or Δλ value was better than 80% correct overall in at least three consecutive nights.

### Experiment 3: Neutral Point

The so-called Neutral Point represents the wavelength of a monochromatic light that is, for a dichromat, indistinguishable from a broadband white light. The exact position of the Neutral Point depends on the spectral composition of the white light. The logic of this experiment was therefore similar to the LCD Colour-Mixing experiment in that we tested whether one colour could be simulated by a combination of others. To allow for switching of the position of the positive stimulus, the fibre tips were mounted in a rotatable bar. In order not to provide any clues to the animals as to whether or not the positive stimulus had moved or stayed on the same side, the bar performed two 90 degree turns before every trial, with the second of these rotations – from the vertical to the horizontal – positioning the lights according to the specific trial. A shutter blocked all light output during switching.

Wallaby Miller was initially trained to prefer monochromatic colour stimuli over the white light. The colour stimuli varied between 490 nm and 540 nm, presented at 10 nm intervals. If wallabies were indeed dichromats, this experiment is effectively a relative colour discrimination task with the choice of colours with a higher M/S cone excitation ratio (compared to the Neutral Point) being rewarded. Wavelengths 483 nm–488 nm (at 1 nm intervals) were included as transfer trials at a rate of one in every three trials as it was not clear how a dichromat would perceive these colours relative to white (Neutral Point), and no choices were rewarded or punished. Data were collected over three consecutive nights.

The wavelength range was then shifted to short wavelengths (450 nm–480 nm) and, at the same time, the training regime changed to reward choices of white over monochromatic colour stimuli. Note that, at this point, a trichromat would have to re-learn that white is now positive whereas before it was negative. A dichromat, on the other hand, could simply continue to choose the colour with the higher M/S cone excitation ratio. Wavelengths 483 nm–488 nm were again treated as transfer trials.

After completion of this task, the regime was changed again to reward of the choice of monochromatic colour stimuli over white, as in the initial training. This time, however, the range of tested wavelengths remained unchanged (450 nm–490 nm). Wavelengths above 484 nm were transfer trials at a rate of one in every four trials. Note that, in this case, both dichromats and trichromats would have to change their decision criteria. After the wallaby had learnt this new task, the wavelength range was finally expanded to include all wavelengths from 430 nm–540 nm. The only way to solve this task is to employ an absolute discrimination rule. Stimuli 481 nm–488 nm were non-reinforced transfer trials as a dichromat may not be able to achieve some of these discriminations.

### Statistics

An online binomial calculator was used (http://stattrek.com/online-calculator/binomial.aspx) to determine if the animal’s response to a specific stimulus pair differed from the 50% chance level. In the analysis of brightness effects, Fisher’s Exact Test was used to determine whether two choice frequencies were significantly different (http://www.langsrud.com/fisher.htm; [18]).

## Results

### Experiment 1: LCD Colour-Mixing

**Figure 3:**
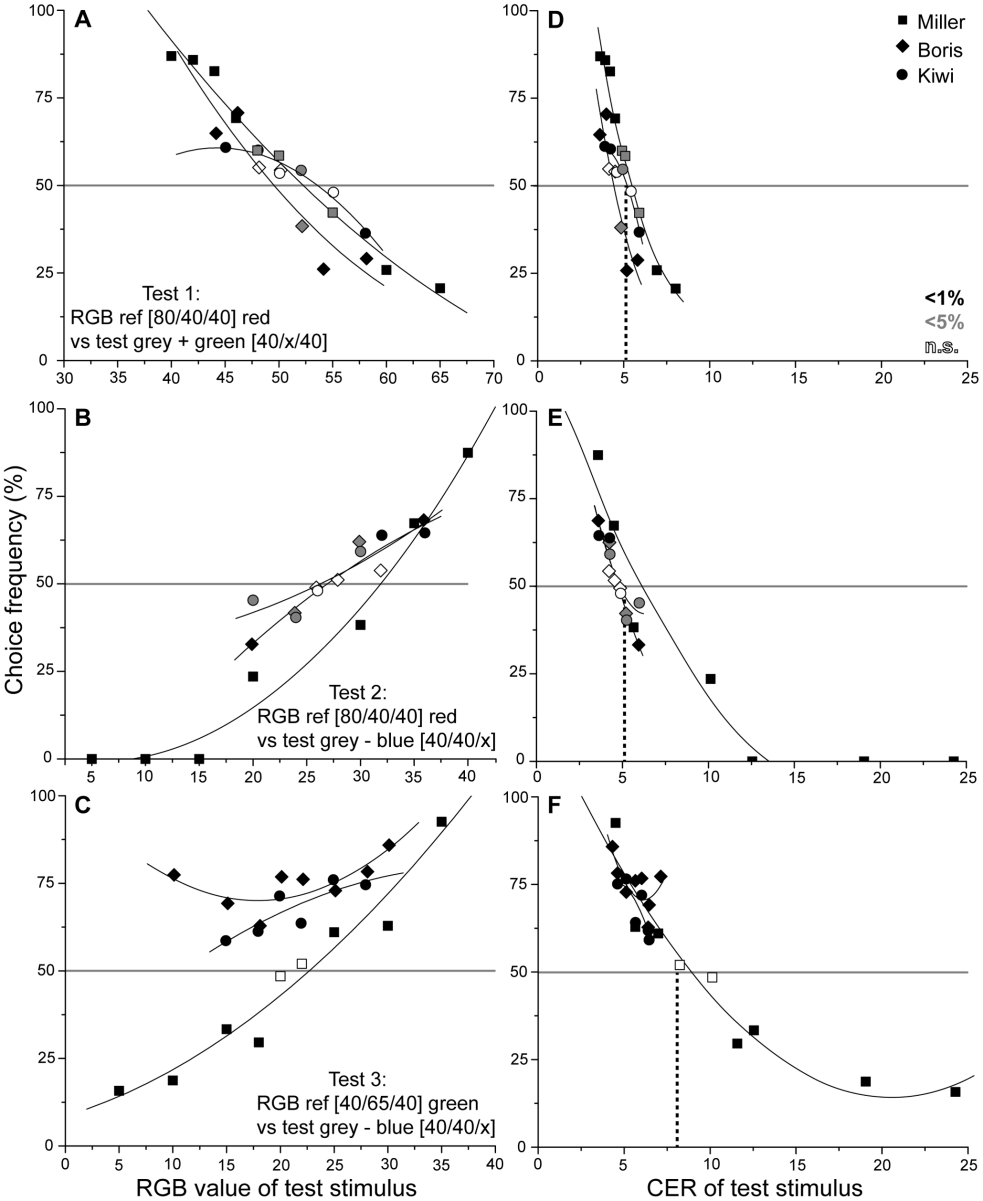
Colour-mixing experiments yielded Confusion Points for various RGB colour combinations. Choice frequencies of the reference stimulus (red in Test 1, A and D; red in Test 2, B and E; green in Test 3, C and F) are presented as a function of a gradually varied colour composition of a test stimulus (green in Test 1, A and D; blue in Test 2, B and E; blue in Test 3, C and F). (A–C) The animals’ stimulus preference reversed, intersecting the 50% mark which was defined as the Confusion Point. (D–E) Converted into excitation ratios of M/S-cones, the data reveal a match between CER for both test and reference stimulus at the Confusion Point (dashed vertical lines).

The cone excitation ratio (CER) of the reference stimulus was calculated from its RGB values and the monitor calibration (Figure 2), and a range of suitable test pairings were determined to include this reference CER. The animals’ responses were analysed and plotted both against the monitor’s LCD values (Figure 3A–C) and against the M/S-CER of the test stimulus (Figure 3D–F).

Having been trained to prefer ‘reddish’ stimuli (RGB = [80/40/40], CER = 5.02) over a grey stimulus (RGB = [40/40/40]; CER = 3.6), discrimination was very high and accurate (squares in Figure 3A,C; 86.93% correct, n = 566, P<0.0001). Discrimination performance of all three animals changed strongly as more green light was added to the grey (Test 1). At either end of the range of test stimuli, responses were significantly different from chance (green LCD values 42, 44, 46: n = 281, all P<0.001; green LCD values 60 and 65: n = 209, P<0.0001), revealing a reversal of stimulus preference from the reference to the test stimulus. At intermediate values of green (LCD values 48, 50, 55: n = 291, P>0.05), choice performance was indistinguishable from chance – with the exception of the first animal tested (Miller) where the test stimuli did not match the Confusion Point and, coincidentally, all results were significantly different from chance. The reversal of the animal’s preference, however, clearly indicates the existence of the Confusion Point. A second order polynomial fit was used to determine its location as the intersection with the 50% mark. This Confusion Point very closely matched the CER of the reddish training stimulus (vertical dashed line in Figure 3D,E), suggesting that the wallabies indeed based their choices on the CER. The same conclusion can be drawn when reducing the blue component of the grey reference light (Test 2) when it is compared against the same ‘reddish’ stimulus (Figure 3B,E). For the condition where blue and green were traded off (Test 3), the reversal was also significant for the one animal tested (Figure 3C,F) at either end of the test stimuli (5, 10, 15, 18: n = 382, P<0.001; 25, 30, 35: n = 224, P<0.04).

Stimuli across the tested colour range differed in brightness but preference of either end of the range did not align with this variation in stimulus brightness. In Test 1, for instance, the preferred green test stimulus was brighter than the red reference, whereas in Test 2, the preferred blue test stimulus was darker than the reference.

All results presented here were obtained from non-reinforced transfer trials.

### Experiment 2: Wavelength Discrimination

**Figure 4:**
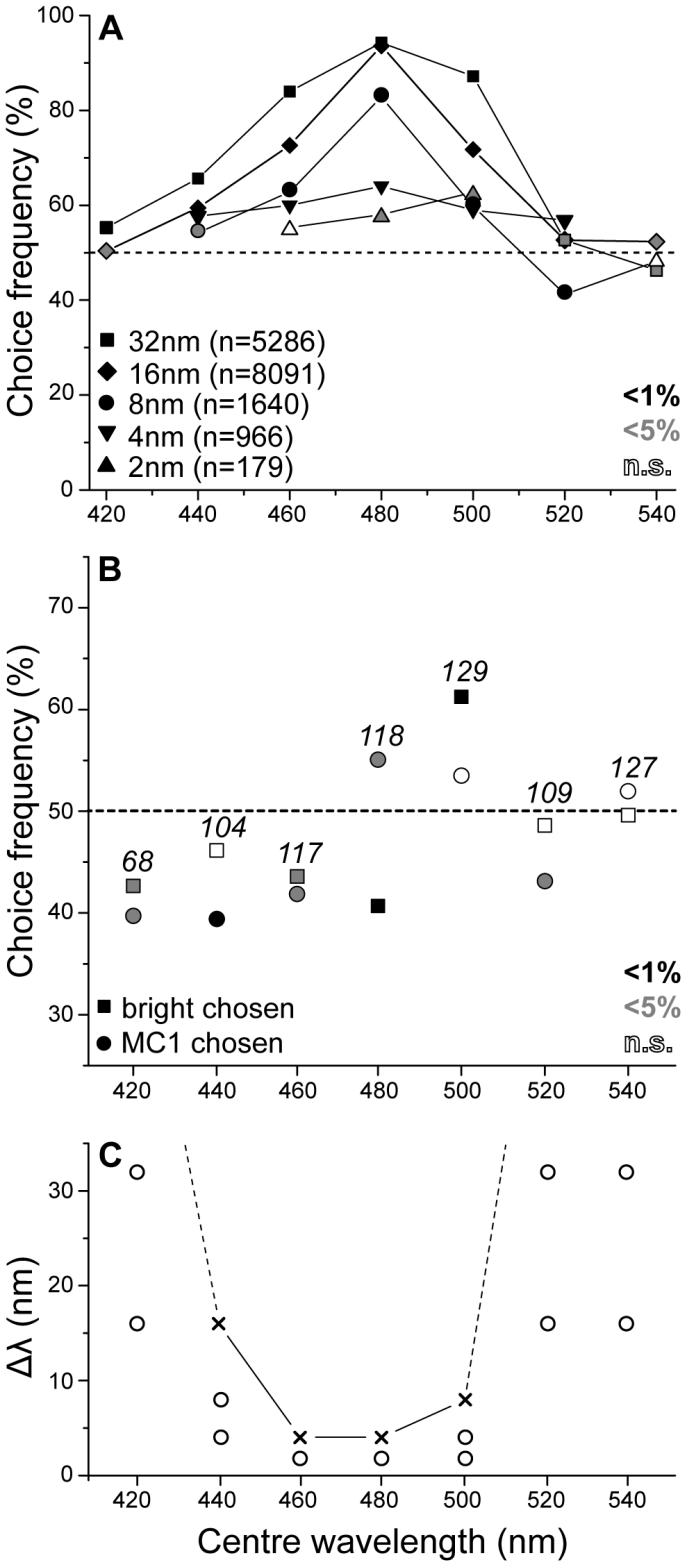
Wavelength discriminability is restricted to a relatively narrow spectral range. (A) For a wallaby, discriminating between two monochromatic stimuli that differed in wavelengths between 2 nm and 32 nm was only reliable in the range between 460 nm and 500 nm where performance consistently exceeded 60% correct responses. Performance was best at centre wavelength 480 nm with significant discrimination ability down to 4 nm wavelength difference, i.e. presentation of the stimulus pair 478 nm *vs* 482 nm. There is an indication at centre wavelengths 480 nm and 500 nm that the wallaby was able to discriminate stimuli that only differed by 2 nm, but these data were based on much smaller sample sizes. (B) Choice frequencies in non-reinforced brightness test trials (Δλ = 0 nm, samples sizes in Italics) were affected by differences in stimulus brightness (squares) and by stimulus presentation by two monochromatic light sources (circles) but did not reveal a consistent pattern across wavelengths. (C) The Minimum Delta Lambda function for the wallaby exhibits a narrow, single-trough shape. The ability to discriminate between lights that differ in wavelength by 16 nm or less was restricted to a narrow spectral range between 440 nm and 500 nm (significant results denoted by crosses, insignificant measurements included as dots).

Data from the Wavelength Discrimination experiment (or ‘Minimum Delta Lambda’) show a clear peak of choice frequency for the positive stimulus around 480 nm (Figure 4A) for all wavelength differences, or delta lambda values (Δλ), except the smallest (Δλ = 2 nm). Discrimination performance was generally better for larger Δλ and worse for the shorter and longer wavelengths of the tested range. Many of the lower choice percentages, however, were still significantly different from chance due to the large sample sizes achieved for some of the pairings (e.g. for Δλ 32 nm at 420 nm: n = 396, P = 0.004; and for Δλ 32 nm at 520 nm: n = 714, P = 0.01). As Δλ between test stimuli decreased from 32 nm to 8 nm, the curves narrowed around peak performance at 480 nm. Discrimination performances at centre wavelengths 480 nm and 500 nm with Δλ = 2 nm were only just significant (n = 47, P = 0.03 and n = 81, respectively; P = 0.02). Sample sizes were much lower because these difficult discriminations were less frequently presented to mitigate frustrating the wallaby.

It is not clear why there is a significant discrimination at centre wavelength 520 nm for stimuli that differed by 8 nm (i.e. 516 nm *vs* 524 nm) where the animal showed a preference for the non-rewarded stimulus (n = 274, P<0.0001).

To prevent the wallaby from exploiting potential small differences in brightness, stimuli were never shown at ‘matched’ brightness, but always differed in brightness by 40% to make it very difficult to use these differences to consistently distinguish between different colours. Control experiments (transfer trials with no difference in wavelength, i.e. Δλ = 0 nm) revealed small effects in the wallaby’s choice performance (Figure 4B), none of which were statistically significant though. These effects were generally less than 10% and not constant across wavelengths. The balanced design of the experiment with respect to brightness meant that positive and negative stimuli were equally often brighter or darker and assured that the brightness bias for some of the stimuli did not significantly contaminate the results (squares in Figure 4B). A small bias may however, remain, possibly explaining some of the small but significant choice performances. Similarly, there was a small and again both inconsistent and insignificant effect with regard to the monochromator that happened to present the stimulus of the wallaby’s choice (circles in Figure 4B).

The wavelength discrimination data shown in Figure 4A were converted to a Minimum-Delta-Lambda function by taking the lowest Δλ at each centre wavelength that still yielded a significant result. At the outer ranges of centre wavelengths where even the largest wavelength difference, Δλ = 32 nm, remained insignificant in our experiment, the curves were extrapolated towards Δλ = 64 nm for illustration purposes. The resulting curve shows a single distinct trough around 460 nm to 480 nm (Figure 4C) where a discrimination ability of Δλ = 4 nm was observed. Outside of this narrow range, discrimination dropped off very steeply.

### Experiment 3: Neutral Point

This experiment was designed to identify whether there is a monochromatic light that wallabies cannot distinguish from a broadband white light. Such a Neutral Point exists only for dichromats (see [16] for a review). The initial training exploited the wallabies’ natural tendency to perform relative discrimination of two colours, in this case with white being the negative stimulus against 480 nm–540 nm monochromatic lights.

**Figure 5:**
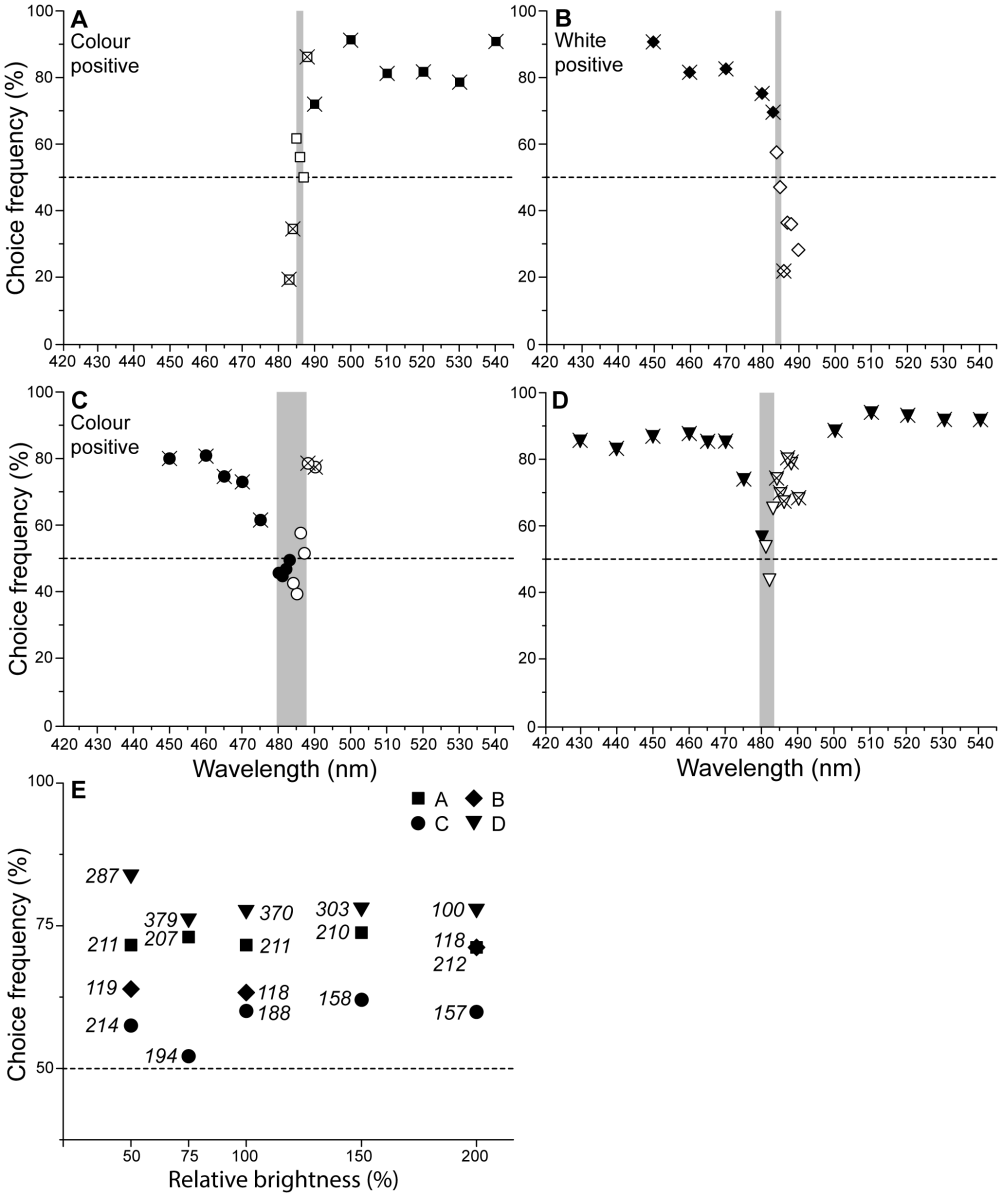
Discriminability in the wallaby shows a Neutral Point where white is indistinguishable from colour stimuli. Open symbols represent non-reinforced transfer trials. Crosses denote statistical significance <5%. (A) Responses when choice of the coloured light was rewarded over the white light for colours above 480 nm (n = 1051). Choices at wavelengths 485 nm–487 nm were not significant, indicated by the grey vertical bar. (B) Responses when the wavelength range was shifted to 450 nm–490 nm and choice of white was rewarded over colour stimuli. Performance was not significant for wavelengths 484 nm and 485 nm, as well as 487 nm, 488 nm, and 490 nm (n = 355). The possible range containing the Neutral Point is indicated by the grey vertical bar. (C) Colour discrimination performance after a reversal in reward rules for the wavelength range 450 nm–490 nm (n = 911). The choice of short-wavelength monochromatic stimuli was rewarded over white. The preference of colours over the white light on both sides of the proposed Neutral Point (grey bar) indicates the use of an absolute colour discrimination rule. (D) Colour discrimination over an extended wavelength range of 430 nm–540 nm where choice of the colour stimuli was rewarded over white. Responses show that monochromatic lights could be discriminated from white light for both short and long wavelength colours (n = 1439), but not for colours around 4850 nm–483 nm. (E) Responses in the Neutral Point experiment were independent of stimulus brightness. Average choice frequencies did not change systematically with the brightness level at which the colour stimulus was presented against the white light in any of the four experimental stages A–D. Italic numbers give sample sizes per data point.

The data from three consecutive nights (Figure 5A) show that discrimination performance was very high for wavelengths above 488 nm (>86% correct; n = 1051, all P<0.0001). For shorter wavelengths, performance dropped dramatically, reaching chance level for wavelengths of 486 nm and 487 nm (56.1% and 50% correct, respectively; P>0.4), before choice preference actually reversed as wavelengths decreased to 485 nm (P = 0.0519), 484 nm (P = 0.0247), and 483 nm (P<0.0001). Stimulus choice was not reinforced at transfer trial wavelengths 483 nm–488 nm. This pattern is consistent with the hypothesis that discrimination is based on the M/S-CER in a dichromatic colour vision system in that the white light would have to excite the cones at a similar ratio as monochromatic wavelengths of 485 nm–487 nm.

The colour range was then shifted to wavelengths below the expected Neutral Point, displaying stimuli between 450 nm–490 nm of which wavelengths 484 nm–490 nm were non-reinforced transfer trials (Figure 5B), with white now being the positive stimulus. To a trichromatic human observer, this effectively meant a reversal from ‘white negative’ to ‘white positive’. For a dichromat, performing a relative colour discrimination task, however, the reward paradigm did not actually change, and the choice of the stimulus that produces a lower M/S-CER still led to correct responses in most cases. Already during the first night of the new task, the animal readily chose (and was rewarded for) white in over 70% of cases (n = 355, P<0.007). Performance was at chance level for transfer trial wavelengths above 484 nm with a trend that colour stimuli above 486 nm were preferred over white (but significant only for 486 nm, P = 0.0075).

In an attempt to train the wallaby to make absolute rather than relative colour discriminations, the reward scheme was reversed such that short wavelength monochromatic colour stimuli were rewarded over white across a wavelength range that included the Neutral Point (450 nm–490 nm) with wavelengths 483 nm–490 nm set as non-reinforced transfer trials. This reversal led to a major decline in the animal’s performance which hardly deviated from the 50% mark for two weeks. Once overall choice frequencies of 70% correct responses were reached again, meaning the task had been learnt, data were collected over three consecutive nights (Figure 5C) in which the wallaby chose all colours both below 475 nm (>61.5% correct responses) and above 488 nm (>77.4% correct responses) when presented against white light (n = 911, P<0.02). Choice frequencies between 480 nm and 487 nm were not significant (39.3%–57.6% correct; P>0.29). Despite four of these stimuli (480 nm–483 nm) being reinforced training trials, requiring the animal to correct an initial wrong choice, performance remained at chance level. This pattern of results is no longer aligned with a relative discrimination strategy but indicative of an absolute discrimination task as colours on both sides of the proposed Neutral Point were preferred over the white light.

Choice frequencies continued to show evidence of absolute discrimination as the tested wavelength range was extended to include longer wavelengths (430 nm–540 nm). The performance curve (Figure 5D), with data collected over six consecutive nights (n = 1439), shows a distinct drop in performance around the predicted Neutral Point. All wavelengths below 475 nm (>74.1% correct responses; P<0.0001) and above 484 nm (>67.4% correct responses; all P<0.04) were successfully discriminated from white. The range containing the Neutral Point, i.e. the wavelength of 50% choice frequency, appears to lie between 480 nm–483 nm.

In the presentation protocol, each monochromatic colour stimulus was included to be presented against the white light at an either darker brightness level (50% or 75%), at matched brightness (100%) or brighter (150% and 200%) than the white equally often. In none of the four stages of the Neutral Point experiment (Figure 5A–D) did the brightness level systematically affect choice frequency of the colour stimuli (Figure 5E), and choice frequencies at different intensities were not significantly different.

This series of experiments provided an indication of the presence of a Neutral Point at 484 nm–487 nm by relative discrimination (Figure 5A,B) or 480 nm–483 nm by absolute discrimination (Figure 5C,D) and, ultimately, of wallabies being dichromats.

## Discussion

The results of all three paradigms, obtained from three animals (two males and one female) on two different experimental setups are clearly consistent with the hypothesis that the tammar wallaby has a dichromatic colour vision system. In Experiment 1– LCD Colour-Mixing, the cone excitation ratio (CER) of two competing stimuli of different colour composition were well predicted and matched a dichromatic colour vision system with spectral sensitivities as previously described for the wallaby [4]–[6]. Experiment 2– Wavelength Discrimination provided the first so-called ‘Minimum Delta Lambda’ function for a marsupial and highlighted the narrow range of good colour discrimination ability in the wallaby, as is typical for a dichromat. The results from Experiment 3– Neutral Point offer the strongest evidence in that we clearly identified a narrow range of wavelengths where white light is not distinguished from a monochromatic colour by the wallaby. The automated nature of the experiments allowed us to produce very high sample sizes for reliable statistical confirmation of our results.

The strength of the LCD Colour-Mixing experiment was the close match of predicted and behavioural Confusion Points. Confusion Points were predicted by converting the M- and S-cone peak spectral sensitivities (M-cones 540 nm and S-cones 420 nm; see [4]) into excitation ratios of these two cone types. The reversal of choice preference aligned closely with the CER of reference and test stimuli in all three stimulus constellations and in all three animals (Figure 3A–F).

For a trichromatic human observer, the colour of the test stimulus in the LCD Colour-Mixing experiment also changed, but it was always distinctly different from the constant reference stimulus, and there was no sense of a Confusion Point or stimulus reversal across the range of test stimuli. Given the low ambient light in the wallaby enclosure at the time of experiment, rod intrusion could have occurred, irrespective of the brightness of the stimulus colours. If a 500 nm pigment had contributed to colour vision, however, the wallabies would have been expected to behave more like a trichromat than a dichromat.

**Figure 6:**
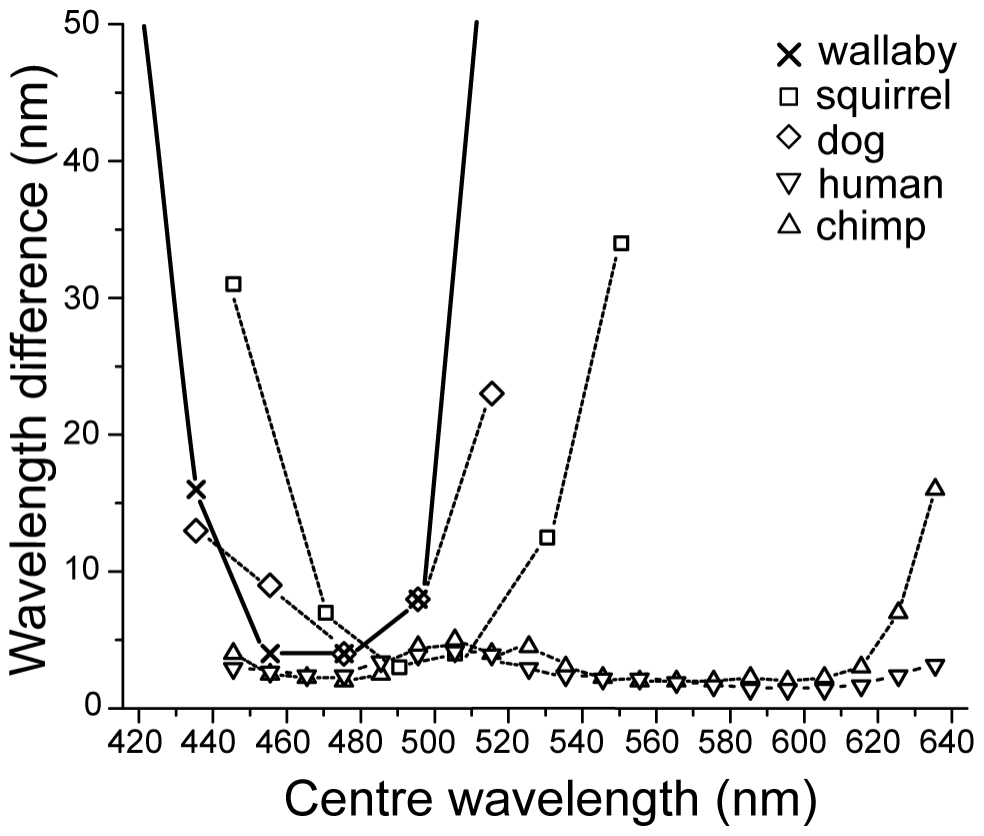
Minimum Delta Lambda functions for five mammalian species show differences between dichromats and trichromats. Trichromatic primates (triangles: [21]) exhibit good colour discrimination ability across a wide wavelength range. In contrast, the data from the wallaby (crosses) much more closely match the discrimination abilities of dichromatic mammals with good performance only over a narrow range of wavelengths (squares: tree squirrel [19]; diamonds: dog [20]).

Our results in the Wavelength Discrimination experiment revealed a narrow range of discriminable wavelengths (440 nm–500 nm; Figure 4A) that strongly suggests wallaby colour vision being based on two, rather than three (or more) cone types. In the peak discriminability range of 460 nm–480 nm, the wallaby was capable of telling apart stimuli differing by 4 nm or even less which is in agreement with [4] who predicted that wallabies were able to discriminate wavelengths of 3 nm difference or less. A limit of about 2 nm also agrees well with the results from the Neutral Point experiment: the width of the performance drop around the Neutral Point was in the order of a 2–3 nm. The accuracy of our monochromatic light sources did not allow us to test differences below 2 nm but performance at 2 nm was already very poor. While the precise characteristics of the ‘Minimum Delta Lambda’ curve remain species-specific, its global shape with a single trough agrees much better with data from dichromatic species (Figure 6; tree squirrel [19]; dog [20]) than with trichromatic primate species [21]. ‘Minimum Delta Lambda’ curves of wallaby, squirrel, and dog all display a narrow range with discrimination ability below Δλ 35 nm for wavelengths 440 nm to 550 nm only, dropping off sharply to either side. In contrast, both humans and chimpanzees are able to reliably discriminate wavelengths above 540 nm and up to 640 nm.

The Neutral Point experiment represents not only an extraordinarily accurate measurement of the dichromatic Neutral Point but also the first attempt to exploit both relative and absolute colour discrimination techniques in the wallaby. Both paradigms yielded virtually identical results. There clearly exists a monochromatic colour that the wallaby was not able to distinguish from the white light. The animal was able to learn and adapt its behaviour according to the task it was given. Having learnt a reversal of preference for the white (Figure 5B), the wallaby managed to switch from a relative discrimination task to an absolute discrimination task (Figure 5C,D), but it was never able to distinguish white light from monochromatic lights between 480 nm–483 nm, despite thousands of trials. In another experiment, not included here, it was attempted to train an animal to a simple absolute colour discrimination, without prior relative discrimination training: the same white light was presented against either a longer-wavelength stimulus (i.e. above the Neutral Point, with a higher cone excitation ratio) or a shorter-wavelength stimulus (i.e. below the Neutral Point, with a lower cone excitation ratio), rewarding the choice of white in both stimulus combinations. The attempt failed, despite hundreds of trials, further supporting the conclusion that for dichromats – while possible – relative colour discrimination was preferred over absolute. The existence of the Neutral Point is a distinct characteristic of the dichromatic colour space (e.g. [16]), however, the precise location is dependent on the spectral properties of the reference broadband stimulus. There is no evidence to assume that the colour at the Neutral Point was of a special quality to the dichromatic observer or that it divides the colour spaces above and below the Neutral Point [4], [22].

Building on a previous experiment with wallabies and confirming its prediction of a Neutral Point between 480 nm–490 nm [4], the current study pin-points the Neutral Point to a very narrow range of indistinguishable wavelengths: 484 nm–487 nm (relative discrimination) or 480 nm–483 nm (absolute discrimination). This compares to Neutral Points of around 500 nm in the tree squirrel [19] and around 480 nm in the guinea pig [23], dog [20], and horse [24], [25]. Variations in the precise location of the Neutral Point are due to the spectral composition of the reference white and the spectral sensitivity of the cone types.

## Conclusions

Our results consistently confirm the tammar wallaby as a dichromat. This matches previous immunohistochemical [5], [9] and behavioural studies [4], despite the fact that its close relative – the quokka – appears to have three different cone types, according to microspectrophotometry and anatomy results [10]. There is also evidence that the common brushtail possum, a nocturnal Australian marsupial more distantly related to the wallaby, is a dichromat with only two cone types identified by immunohistochemistry (L. Vlahos; unpublished data). These results are further in agreement with data from South-American opossums – marsupials that branched off very early within the lineage – that strongly suggest these species, too, to only have two cone types [3], [14]. In the absence of molecular evidence for a third cone opsin class [9], [12], [13], [15] and given the uncertainty of rod intrusion having potentially confounded the behavioural experiments in the fat-tailed dunnart [11], there is a definite need to further investigate the colour vision abilities of Australian marsupials by replicating the behavioural experiments with the dunnart and extending these to other marsupials. This will clarify whether these animals are indeed cone trichromats or not.
